# Characteristics of United States military pilots with atrial fibrillation and deployment and retention rates

**DOI:** 10.1186/s12872-022-02542-8

**Published:** 2022-03-13

**Authors:** Andrea N. Keithler, Andrew S. Wilson, Alexander Yuan, Jose M. Sosa, Kelvin N. V. Bush

**Affiliations:** Division of Cardiology, Brooke Army Medical Center, 3551 Roger Brooke Drive, San Antonio, TX 78234 USA

**Keywords:** Atrial fibrillation, Arrhythmia, Pilots, Military, Ablation, Pulmonary vein isolation

## Abstract

**Introduction:**

Atrial fibrillation (AF) is an arrhythmia that impacts deployment and retention rates for United States military pilots. This study aims to characterize United States active duty (AD) pilots with AF and review deployment and retention rates associated with medical and ablative therapies.

**Methods:**

An observational analysis was performed to assess AD pilots diagnosed with AF in the largest military regional healthcare system from 2004 to 2019. Baseline characteristics and AF management were reviewed.

**Results:**

27 AD pilots (mean age, 37.3 ± 7.9 years; mean BMI, 27.3 ± 3.1 kg/m^2^; 100% male sex) were diagnosed with AF during the study dates. 17 (63%) were Air Force branch pilots with hypertension as the most common risk factor (26%). There were overall low CHA_2_DS_2_-VASc scores (mean 0.29 ± 0.47). 22 (82%) pilots were equally treated with medical rate and rhythm strategies (41% and 41%, respectively). 16 (59%) underwent pulmonary vein isolation (PVI) with zero complications. 11 (41%) pilots received warfarin and 5 (19%) received a direct oral anticoagulant for stroke prevention. After diagnosis, 12 (44%) pilots deployed and 25 (93%) were retained in military. PVI was not associated with a change in subsequent deployments rates (PVI, 38% vs no PVI, 55%; *p* = 0.3809) or retention rates (PVI, 94% vs no PVI, 91%; *p* = 0.7835).

**Conclusions:**

United States military pilots diagnosed with AF are younger patients with few traditional AF risk factors and  they receive medical rate and rhythm strategies equally. Many pilots maintain deployment eligibility and most remain on AD status after diagnosis. PVI is not associated with differences in retention or deployment rates. Further prospective study is needed to further evaluate these findings.

## Introduction

Atrial fibrillation (AF), a cardiac rhythm disorder commonly associated with increasing age [1], is now readily recognized to occur with fivefold greater lifetime risk in athletic individuals compared to sedentary populations [2]. A chronic hypertension diagnosis only imparts a 1.42-fold increased risk of AF as the most common traditional underlying risk factor [3, 4]. The pathophysiology of AF in athletes and other similar populations is incompletely understood, but is theorized to be related to large fluctuations in vagal tone, atrial stretching mechanisms, and increased inflammation associated with high levels of endurance exercise [2]. The prevalence and epidemiology of AF in the athletic population has been predominately drawn from retrospective and case control studies and the literature lacks prospective studies carefully quantifying exercise exposures in this population [5–10]. Identified risk factors for AF within the athletic population include male sex, middle age, tall stature, participation in endurance athletics, lifetime exercise of greater than 1500–2000 h, and high occupational physical activity [1, 11–13].

Active duty (AD) military personnel and military pilots are mandated to maintain a high level of physical fitness compared with the general population to fulfill their occupational duties and have similar physical profiles to athletes diagnosed with AF as described in the literature [14–16]. In addition to elevated physical fitness standards compared to the average individual, military pilots are specifically exposed to other unique and known precipitants of AF: reduced partial pressures of oxygen at high altitudes, sustained acceleration and gravitational forces, and physical and emotional stress related to combat flying [17, 18]. AF can be highly symptomatic and potentially incapacitating with resultant dire consequences for pilots while operating aircrafts [17, 18]. Furthermore, AF can be a grounding medical condition for AD pilots with substantial global impacts on military deployments, readiness, and retention [19, 20]. The diagnosis of AF in itself does not automatically preclude continued military service provided that the individual continues to maintain fitness for general military duty requirements.

The AF treatment approach in military pilots has been traditionally individualized in accordance with guideline recommendations [21]. There is currently no clear and documented consensus on the appropriate management or military disposition for AD pilots with AF diagnoses as there are no prior reports on their demographics or historical military dispositions. This lack of standardized management is further compounded by the current broader discussions and controversies surrounding medical rate and rhythm strategies in the general population [1]. Catheter ablation is an alternative and invasive option demonstrated to be effective in eliminating AF and improving exercise tolerance in several small studies of endurance athletes but the safety and utilization in military pilots has not been previously described [22–24]. There are currently no reports describing these treatment approaches in military pilots diagnosed with AF. This study aims to describe United States AD military pilots diagnosed with AF and characterize the long-term outcomes of deployment and military retention associated with the medical and ablative therapies received.

## Methods

### Study design

AD military pilots within the San Antonio Military Health System (United States Air Force, Army, and Navy services) who were diagnosed with AF between January 1, 2004 to July 31, 2019 (n = 27) were included and retrospectively reviewed. Eligibility criteria included AD status at time of diagnosis, occupation, and confirmed 12-lead electrocardiographic diagnosis of AF by a board-certified cardiologist. Study patients were symptomatic or asymptomatic. Retired military, military dependents, and service members with an unknown occupation were excluded. Distribution of baseline characteristics, medical therapies, and deployment and retention rates were evaluated by electronic medical record and medical board documentation review. An individual was qualified as receiving anticoagulation if they had a history of a prescription for an anticoagulant. Service member retention was defined as someone who was not discharged from the service and remained on AD status throughout the duration of the study. Military members were classified as serving a deployment if they were mobilized for determined mission critical purposes or duty tours.

### Statistical methods

The association between management therapies, future deployments, and military disposition was analyzed using Chi Square and Fisher’s Exact Test for categorical variables. The T-Test was used for comparison of continuous variables. Due to the sample size, we relied on central limit theorem and did not test normality assumption. This research protocol was approved by the institutional review board of the San Antonio Military Medical Center. The institutional review board of San Antonio Military Medical Center waived the need for informed consent because the research involved no more than minimal risk to the participants and the waiver did not adversely affect their rights and welfare. Data analysis was performed using JMP statistical analysis software Version 15 (SAS Institute, Cary, NC).

### Patient and public involvement

Patients and the public were not directly involved in the research process given the retrospective, observational nature of the study.

## Results

There were 27 male AD pilots with a mean age of 37.3 ± 7.9 years, mean BMI of 27.3 ± 3.1 kg/m^2^ and predominately Caucasian race (85%) (Tables 1, 2). 17 (63%) were Air Force pilots and the majority had paroxysmal AF (93%). There were no pilots who were determined to have permanent AF. Hypertension and obstructive sleep apnea were present in 26% and 19%, respectively. None of the pilots carried diagnoses of diabetes mellitus, vascular disease, transient ischemic attack (TIA), cerebrovascular accident (CVA), or coronary artery disease. There was a collective mean CHA_2_DS_2_-VASc score of 0.29 ± 0.47 and a mean HAS-BLED score of 0.74 ± 0.53. The cohort overall had few cardiovascular risk factors and no known history of underlying cardiovascular disease (Tables 1, 2). After AF diagnoses, 12 (44%) pilots completed deployments and 25 (93%) were retained on AD military status (Table 3). Two members separated or retired, both for reasons unrelated to AF. Two pilots with persistent AF were retained but never deployed after their diagnosis.

**Table 1:** Demographic characteristics of active duty military pilots diagnosed with atrial fibrillation and relationship to medical treatment strategy

	All (n = 27)	No medical therapy (n = 5)	Rate control (n = 11)	Rhythm control (n = 11)	*p* value
Age, years	37.3 ± 7.9	29.2 ± 6.2	40.3 ± 7.2	38.0 ± 7.1	0.0147
Male, no. (%)	27 (100%)	5 (100%)	11 (100%)	11 (100%)	N/A
BMI, kg/m^2^	27.3 ± 3.1	25.0 ± 3.8	28.8 ± 3.1	26.9 ± 1.8	0.1408
Caucasian, no. (%)	23 (85%)	5 (100%)	7 (64%)	11 (100%)	N/A
African American, no. (%)	1 (4%)	0 (0%)	1 (9%)	0 (0%)	N/A
Other race/unknown race, no. (%)	3 (11%)	0 (0%)	3 (27%)	0 (0%)	N/A
Hypertension, no. (%)	7 (26%)	1 (20%)	3 (27%)	3 (27%)	0.9431
Obstructive sleep apnea, no. (%)	5 (19%)	1 (20%)	2 (18%)	2 (18%)	0.9956
History of heart failure, no. (%)	1 (4%)	0 (0%)	1 (9%)	0 (0%)	0.3961
Glucose, mg/dL	92.1 ± 11.5	93.8 ± 15.4	92.5 ± 8.5	91.1 ± 13.3	0.3401
Total cholesterol, mg/dL	187.5 ± 27.0	194.2 ± 17.6	189.5 ± 34.9	182.5 ± 22.2	0.2335
LDL, mg/dL	117.8 ± 22.7	108.3 ± 21.1	123.3 ± 27.8	115.8 ± 17.7	0.3409
HDL, mg/dL	47.7 ± 9.9	57.1 ± 13.7	45.2 ± 5.8	46.0 ± 2.9	0.0612
Triglycerides, mg/dL	115.6 ± 82.2	148.0 ± 156.7	110.9 ± 69.6	105.5 ± 47.3	0.8181
CHA_2_DS_2_-VASc score	0.29 ± 0.47	0.20 ± 0.45	0.36 ± 0.50	0.27 ± 0.47	0.5732
HAS-BLED score	0.74 ± 0.53	0.80 ± 0.45	0.64 ± 0.67	0.82 ± 0.40	0.3706
AF classification					
Paroxysmal AF, no. (%)	25 (93%)	5 (100%)	11 (100%)	9 (82%)	N/A
Persistent AF, no. (%)	2 (7%)	0 (0%)	0 (0%)	2 (18%)	N/A

Values are number (%), mean (± 1 standard deviation)

*AF* atrial fibrillation, *BMI* body mass index (calculated as kg divided by meters squared), *CVA* cerebrovascular accident, *HDL* high density lipoprotein, *LDL* low density lipoprotein, *N/A* not applicable, *TIA* transient ischemic attack

**Table 2:** Demographic characteristics of active duty military pilots diagnosed with atrial fibrillation who underwent pulmonary vein isolation compared to pilots without pulmonary vein isolations

	All (n = 27)	No PVI (n = 11)	PVI (n = 16)	*p* value
Age, years	37.3 ± 7.9	34.6 ± 7.9	39.1 ± 7.5	0.1519
Male, no. (%)	27 (100%)	11 (100%)	16 (100%)	N/A
BMI, kg/m^2^	27.3 ± 3.1	26.3 ± 3.4	28.0 ± 2.7	0.2564
Caucasian, no. (%)	23 (85%)	10 (91%)	13 (81%)	N/A
African American, no. (%)	1 (4%)	0 (0%)	1 (6%)	N/A
Other race/unknown, no. (%)	3 (11%)	1 (9%)	2 (13%)	N/A
Hypertension, no. (%)	7 (26%)	2 (18%)	5 (31%)	0.9024
Obstructive sleep apnea, no. (%)	5 (19%)	2 (18%)	3 (19%)	0.9702
History of heart failure, no. (%)	1 (4%)	1 (9%)	0 (0%)	0.1735
Glucose, mg/dL	92.1 ± 11.5	93.0 ± 11.5	91.6 ± 11.9	0.7295
Total cholesterol, mg/dL	187.5 ± 27.0	189.1 ± 21.0	186.4 ± 31.0	0.7671
LDL, mg/dL	117.8 ± 22.7	115.0 ± 17.0	119.6 ± 26.0	0.9790
HDL, mg/dL	47.7 ± 9.9	49.0 ± 12.8	46.9 ± 7.6	0.9803
Triglycerides, mg/dL	115.6 ± 82.2	130.5 ± 120.0	105.4 ± 42.9	0.6568
CHA_2_DS_2_-VASc score	0.29 ± 0.47	0.27 ± 0.47	0.31 ± 0.48	0.8273
HAS-BLED score	0.74 ± 0.53	0.91 ± 0.54	0.63 ± 0.50	0.1875
AF classification				
Paroxysmal, no. (%)	25 (93%)	10 (91%)	15 (94%)	N/A
Persistent, no. (%)	2 (7%)	1 (9%)	1 (6%)	N/A

Values are number (%), mean (± 1 standard deviation)

*AF* atrial fibrillation, *BMI* body mass index (calculated as kg divided by meters squared), *CVA* cerebrovascular accident, *HDL* high density lipoprotein, *LDL* low density lipoprotein, *N/A* not applicable, *PVI* pulmonary vein isolation, *TIA* transient ischemic attack

**Table 3:** Military retention and deployment rates in active duty pilots diagnosed with atrial fibrillation comparing medical and ablative treatment strategies

	All (n = 27)	Retained (n = 25)	Discharged (n = 2)	Deployed (n = 12)	Non-deployed (n = 15)
Medical therapies for AF					
No medical therapy	5 (19%)	5 (20%)	0 (0%)	5 (42%)	0 (0%)
Rate control agent	11 (41%)	9 (36%)	2 (100%)	4 (33%)	7 (47%)
Rhythm control agent	11 (41%)	11 (44%)	0 (0%)	3 (25%)	8 (53%)
Rates of PVI for AF					
No PVI	11 (41%)	10 (40%)	1 (50%)	6 (50%)	5 (33%)
PVI	16 (59%)	15 (60%)	1 (50%)	6 (50%)	10 (67%)
Anticoagulation management for AF					
No anticoagulation	11 (41%)	11 (44%)	0 (0%)	6 (50%)	5 (33%)
Warfarin	11 (41%)	10 (40%)	1 (50%)	5 (42%)	6 (40%)
Direct oral anticoagulants	5 (19%)	4 (16%)	1 (50%)	1 (8%)	4 (27%)

Values are number, (%)

*AF* atrial fibrillation, *PVI* pulmonary vein isolation

**Fig. 1:**
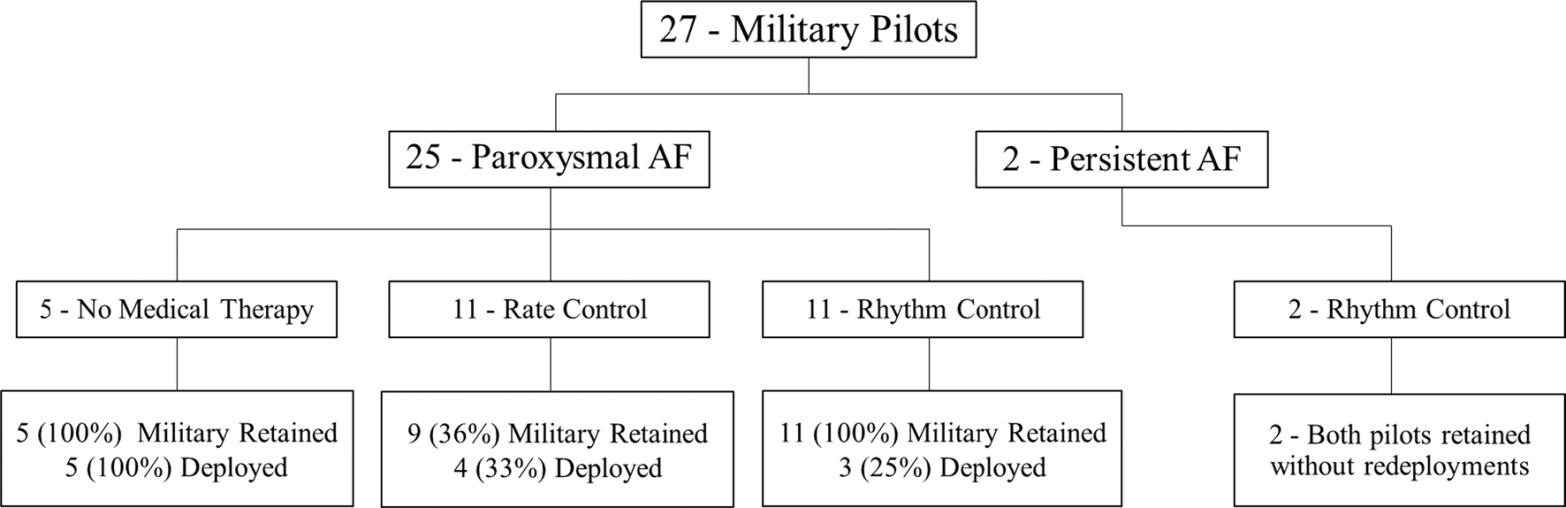
Flow diagram illustrating distribution of paroxysmal and persistent AF with different treatment strategies

A total of 22 (82%) of pilots evenly received rate and rhythm medications (41% and 41%, respectively; Fig. 1). Rate and rhythm medical treatment strategies were not different in their association with deployment rates (rate control, 36% vs rhythm control, 27%; *p* = 0.6467). Pilots who did not receive any medical therapy had 100% deployment rates and this was statistically significant when compared to pilots treated with rate control (*p* = 0.0288) or rhythm control agents (*p* = 0.0128). There was no significant difference in retention rates amongst the treatment groups (no medical therapy, 100% vs rate control, 82% vs rhythm control, 100%; *p* = 0.1475).

**Fig. 2:**
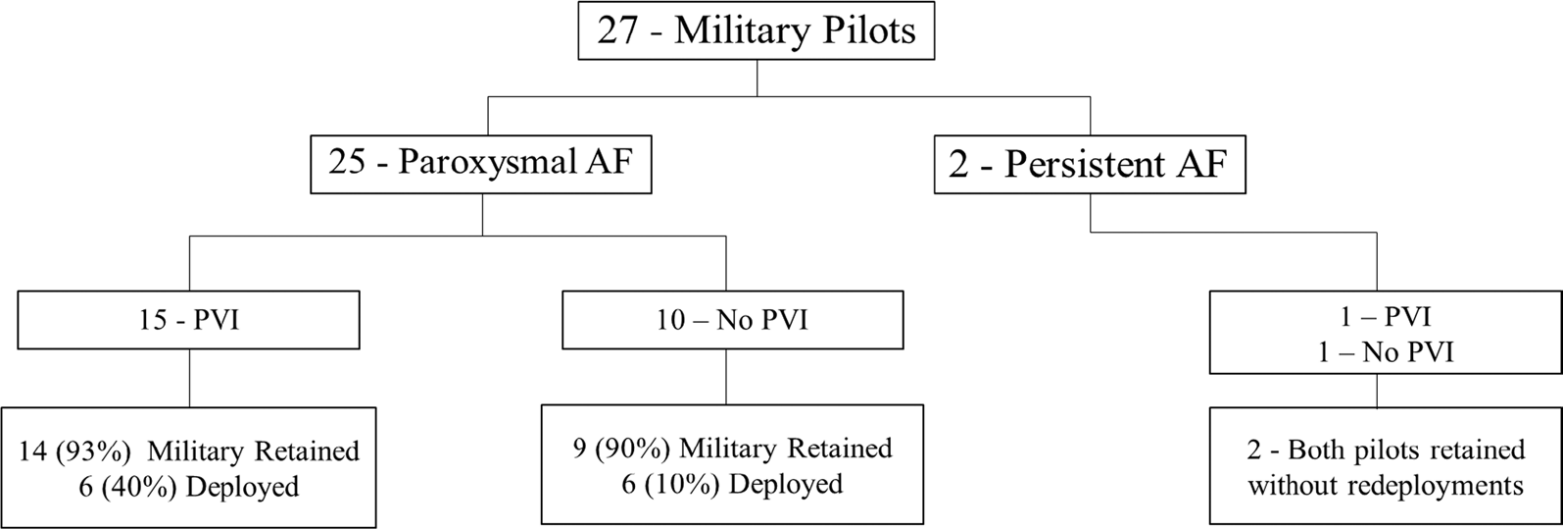
Flow diagram illustrating distribution of paroxysmal and persistent AF pilots receiving PVI

There were 16 (59%) participants who underwent pulmonary vein isolation (PVI) without complications. Of these, six underwent repeat PVI for AF recurrence. PVI was not associated with a change in subsequent deployment rate (PVI, 38% vs No PVI, 55%; *p* = 0.3809) or retention rate (PVI, 94% vs no PVI, 91%; *p* = 0.7835; Fig. 2).

11 (41%) pilots previously received warfarin and 5 (19%) previously received a direct oral anticoagulant (DOAC) for chronic stroke prevention or transient periprocedural anticoagulation. There were no reported strokes in this study group nor bleeding complications in pilots who previously received systemic anticoagulation. There were no differences in deployment rates between prior anticoagulation strategies (no anticoagulant therapy, 55% vs warfarin, 45%, vs DOAC 20%; *p* = 0.4116), however there was a non-statistically significant trend toward fewer deployments in those treated with DOACs. There was no significant difference in military dispositions in association with prior anticoagulation strategy, with most pilots retained across all groups (no anticoagulant, 100% vs warfarin, 80% vs DOAC, 91%; *p* = 0.2790).

## Discussion

This is the first report describing AF management in United States AD military pilots; a predominately younger cohort with few AF or cardiovascular risk factors. AD pilots had low estimated stroke risks by CHA_2_DS_2_-VASc scores and low estimated bleeding risks by HAS-BLED scores.

In other cases of recurrent AF, flying low-performance aircraft operations has been offered following comprehensive evaluation and observation after 3–6 months. For a pilot to return with recurrent, paroxysms of AF they must have adequate rate control achieved with or without medical therapy. There have been no documented cases of recurrent paroxysmal or persistent AF where a pilot has been allowed to return to single seat flying operations or high-performance flying to our knowledge.

Overall, many military pilots were able to complete deployments and met military retention standards after AF diagnosis, 44% and 93%, respectively. A significant proportion of pilots were not deployed following diagnosis, which may indicate uncontrolled symptoms and/or use of medications which were prohibitive to deployment to an austere environment. Only two patients in the cohort did not remain on AD status for non-medical reasons unrelated to their AF diagnosis. When AD pilots were observed to receive no medical or ablative therapies and had no prior history of anticoagulation there were 100% deployment and retention rates; strongly reflective of a low symptom burden of AF and low impact on completing occupational duties. Pilots who remained on active duty service but no longer on flying status had a comprehensive medical evaluation board that determined them unfit for flying but medically capable of performing an alternate primary occupation. The reasons for continued non-flying service could have been related to medical therapy (rate control agents or antiarrhythmics) or symptoms not tolerated.

Just over half of the study pilots were previously treated with anticoagulants after diagnosis; predominantly warfarin and some with DOACs. While many patients had a history of anticoagulation prescriptions, this does not imply they were maintained on an anticoagulant throughout the study period. Data regarding the duration of anticoagulant use is not available as part of this observational study data. We strongly suspect that the indication for systemic anticoagulation was largely driven by procedural interventions such as cardioversions and ablations instead of chronic stroke prevention given the overall low mean CHA_2_DS_2_-VASc of this cohort. Further, the observed association of anticoagulation utilization and deployments in this observational study is limited without clear temporal relationships available. Hence, we would not conclude that our results are suggestive of military pilots being universally cleared, safe or automatically eligible to deploy to all austere conditions while on systemic anticoagulation but instead provide evidence of some pilots being treated for a duration of time and still having capacity to deploy in the future.

Military pilots in this study received medical rate and rhythm control strategies equally. Rate and rhythm control agents were not associated with significant differences in retention or deployment rates. Absence of pharmacologic therapy was associated with a greater deployment rate when compared with those who received rate or rhythm medications and this may be reflective of those particular pilots having a low baseline AF burden and symptomology not requiring any medical therapy as previously discussed.

PVIs were performed in nearly 60% of patients without complications. 6 of 16 underwent repeat PVI for AF recurrence, which might be explained by some theories. First, this could be reflective of aggressive efforts to maintain sinus rhythm and preserve flying status in light of AF recurrences. Second, this could be suggestive of substantial joint efforts between patients and providers to avoid medical therapy and the entailing side effect profiles. PVI did not significantly influence deployment or retention rates in this small cohort but this data does highlight the safety of the procedure in special, younger population as there were zero complications. This study was not specifically designed to evaluate the efficacy of PVI for AF in military pilots but does bring to question the PVI efficacy of reducing AF burden and morbidity outcomes in the military pilot population.

Limitations of this study include the lack of diversity in patients, small cohort size, and retrospective constraints. The evaluation of symptoms throughout the study is also limited. Further, this retrospective review allows us only to draw associations between therapies and outcomes without concluding direct cause and effect relationships. The small number of pilots reviewed in the study is likely a reflection of currently decentralized management of AF and the lowered flight tempo operations in San Antonio. The 2004–2019 dates were chosen as inclusion dates as these were timelines that provided accurate and available electronic medical record data for the small pilot cohort review. The broad range of study dates could have subjected our patients to temporally different guideline treatment strategies; however, there has never been any clear consensus on the management of AF in this special pilot population in the military or civilian medical systems. Despite these limitations we feel the data presented in this study offer value to military and civilian providers given the paucity of literature surrounding military pilots.

There is little data to guide management decisions in young, AD United States military pilots. No prior studies describe pharmacologic treatment strategies or PVI in this unique, mission critical population. This study highlights the need for further research on the efficacy of AF therapies on symptomatology and recurrences in military pilots along with other younger demographic profiles in high-risk occupations.

## Conclusion

United States military pilots diagnosed with AF are younger patients with few traditional AF risk factors. In this small cohort they receive medical rate and rhythm strategies equally, maintain deployment eligibility, and most remain on AD status after diagnosis. PVI is not associated with changes in retention or deployment rates. Multicenter prospective studies are needed to better characterize the appropriate management of AF in the military pilot population.

## Data Availability

The datasets used and/or analyzed during the current study available from the corresponding author on request. All methods were performed in accordance within the guidelines and regulations of the approved research protocol. The research protocol was approved by the institutional review board of the Brooke Army Medical Center. The full dataset is available from the corresponding author (kelvin.n.bush.mil@mail.mil) upon request.

## Abbreviations

AD: Active duty
AF: Atrial fibrillation
BMI: Body mass index
CVA: Cerebrovascular accident
DOAC: Direct oral anticoagulant
HDL: High density lipoprotein
LDL: Low density lipoprotein
N/A: Not applicable
PVI: Pulmonary vein isolation
TIA: Transient ischemic attack
